# The association between uncertainty intolerance, perceived environmental uncertainty, and ego depletion in early adulthood: the mediating role of negative coping styles

**DOI:** 10.3389/fpsyg.2024.1228966

**Published:** 2024-04-11

**Authors:** Xiao Li, Jingjing Song

**Affiliations:** Institute of Psychology, School of Education, China University of Geosciences, Wuhan, Hubei, China

**Keywords:** uncertainty intolerance, perceived environmental uncertainty, negative coping styles, ego depletion, self-control

## Abstract

**Introduction:**

Uncertainty intolerance and perceived environmental uncertainty can influence an individual’s emotions and behavioral responses. Previous research showed that high uncertainty intolerance and high perceived environmental uncertainty were both negatively associated with an individual’s life satisfaction. We explored the interaction effects of uncertainty intolerance and perceived environmental uncertainty on ego depletion of early adulthood and its mechanisms.

**Methods:**

Investigating 292 college students using an uncertainty intolerance scale, a perceived environmental uncertainty scale, a negative coping style questionnaire, and an ego depletion scale. The correlations among all variables were calculated using Pearson’s product-moment correlation coefficient, and then we used the PROCESS macro (model 8) in SPSS to test the conditional process model in the relationship between uncertainty intolerance and ego depletion.

**Results:**

The results showed that the interaction terms of uncertainty intolerance and perceived environmental uncertainty were significantly associated with negative coping styles. Only in the high perceived environmental uncertainty situations, uncertainty intolerance was positively associated with negative coping styles, and negative coping styles were positively associated with ego depletion.

**Discussion:**

In general, compared with perceived environmental uncertainty, participants’ cognition towards environmental uncertainty was much more associated with individual’s coping styles and psychological state, individuals with high uncertainty intolerance would face great stress and experience more emotional problems. Our results suggest that it is important for individuals’ mental health to gain a sense of control in an uncertain environment and improve the tolerance of uncertainty. Future research needs to pay attention to the intervention strategy of decreasing uncertainty intolerance.

## 1 Introduction

Perceived environmental uncertainty refers to an individual’s perception of rapid and drastic environmental changes, and the perception that it is difficult to accurately measure and predict environmental changes (McKelvie et al., 2011; Laguir et al., 2022; Pashutan et al., 2022; Yang et al., 2022). Uncertainty reduction theory posits that environmental uncertainty is a stressful and threatening situation that decreases an individual’s life satisfaction and damages interpersonal relationships; people tend to reduce uncertainty by seeking more information, expending more effort, or making quick decisions to obtain certain results (Knobloch, 2015). The contextual relational uncertainty model further suggests that people’s attitudes and cognition of environmental uncertainty directly affect individual emotions and behavioral responses (Monk and Ogolsky, 2019). Uncertainty intolerance refers to an individual’s excessive tendency to consider it unacceptable that a negative event may occur, however small the probability of its occurrence (Birrell et al., 2011). Previous studies have demonstrated that individuals with higher uncertainty intolerance experience higher anxiety, lower happiness, and lower life satisfaction (Mallett et al., 2021), and are more likely to suffer from sleep disorders (Sabouri et al., 2016). Both uncertainty intolerance and perceived environmental uncertainty would increase ego depletion. Ego depletion refers to psychological exhaustion after completing a task that expends self-control (Baumeister and Vohs, 2016; Friese et al., 2019). This study focused on the association between uncertainty intolerance, perceived environmental uncertainty, and ego depletion, and further explored its internal mechanisms. We believe our results can help identify effective measures to decrease the negative effects of uncertainty intolerance and perceived environmental uncertainty.

### 1.1 The relationship between uncertainty intolerance and ego depletion

Uncertainty intolerance is a stable individual difference in cognitive, emotional, and behavioral tendencies when faced with uncertain situations (Carleton, 2016). Individuals with different levels of uncertainty intolerance have different perceptions, understandings, and experiences of uncertain environments, which further affects their emotional responses and coping styles to uncertain states. The cognitive model of generalized anxiety disorder proposes that intolerance of uncertainty induces worry and anxiety and leads to poor problem orientation and cognitive avoidance (Birrell et al., 2011). Individuals with a low level of uncertainty intolerance may prefer to take risks, experience uncertainty, and be willing to cope with a variety of uncertain situations. Whereas, individuals with a high level of uncertainty intolerance have cognitive bias and attentional bias toward uncertainty, and are unable to tolerate uncertainty in their lives, even when events associated with such uncertainty are not happening (Freeston et al., 2020), tend to regard ambiguous situations as threats, experience nervous and stressed (Yang and Li, 2023). They will expend more concentration, willpower, and other mental strategies to reduce uncertainty.

Previous research has demonstrated that uncertainty intolerance leads to higher anxiety, lower happiness, and life satisfaction (Mallett et al., 2021; Yang and Li, 2023), lower cognitive flexibility (Yildiz and Eldeleklioglu, 2021), more sleep disorders (Sabouri et al., 2016), and greater job uncertainty (López et al., 2022). Asmundson and Taylor (2020) found that high uncertainty intolerance during the COVID-19 pandemic led to excessive anxiety, excessive use or avoidance of medical services, hoarding of medical supplies, distrust of relevant experts and professional information, and overloading of negative information from the Internet.

Previous research has demonstrated that uncertainty intolerance had a significant positive predictive effect on college students’ self-regulatory fatigue, which is positively associated with ego depletion (Wang et al., 2024). Ego depletion refers to the phenomenon that exertion of self-control in a first task leads to impaired subsequent self-control performance compared with a control group that did not exert as much self-control in the first task (Baumeister and Vohs, 2016; Friese et al., 2019). Based on the review above, we hypothesized that uncertainty intolerance was positively associated with ego depletion.

### 1.2 The relationship between perceived environmental uncertainty and ego depletion

Perceived environmental uncertainty has two dimensions: perceived environmental harshness and perceived unpredictability. Specifically, perceived environmental harshness refers to an individual’s perception of the external environment (uncontrollable events, such as wars, diseases, and natural disasters) that cause illness and death (Ellis et al., 2009). Perceived environmental unpredictability is defined as the individual’s perception of the variability and unpredictability of the environment (Behar-Zusman et al., 2020).

People have a basic need to control their lives and gain security. Perceived environmental uncertainty reduces an individual’s sense of control and security, with multiple negative effects on individual emotions and behaviors (Karataş and Tagay, 2021). Previous research suggests that perceived environmental uncertainty increases job insecurity, decreases enthusiasm for work (Hui and Lee, 2000), reduces job satisfaction, and increases turnover intention (Cullen et al., 2014). The COVID-19 pandemic has increased social uncertainty about the economy, employment, personal finances, and interpersonal relationships (Karataş and Tagay, 2021; Evli and Şimşek, 2022). Many studies have also focused on the impact of environmental changes resulting from the COVID-19 pandemic on people’s psychological and behavioral responses. Studies have shown that, at the peak of the pandemic, medical workers who were unsure whether they were infected with COVID-19 experienced more distress and anxiety, leading to lower job satisfaction. The uncertainty caused by the pandemic increased people’s psychological uncertainty (Mallett et al., 2021), negatively affected individual mental health, and reduced life satisfaction and happiness (Mallett et al., 2021; Evli and Şimşek, 2022).

Previous research has demonstrated that unpredictable environment and uncontrollable environment both reduce self-control resources and cause ego depletion (Muraven and Baumeister, 2000; Milkman, 2012). If the current environment is uncertain and does not provide us with information about future events and their outcomes (Laguir et al., 2022), individuals will tend to feel threatened and pressured, and use more cognitive resources to understand and reduce environmental uncertainty, which leads to lower self-control and increased anxiety and depression. A lack of self-control and an increase in negative emotions hinder individuals from making positive and accurate judgments, resulting in a substantial disconnection between their desires and their current state. Therefore, we hypothesized that perceived environmental uncertainty was positively associated with increased ego depletion.

### 1.3 The association between uncertainty intolerance, perceived environmental uncertainty, and ego depletion

To our knowledge, no studies explored the interaction effect of perceived environmental uncertainty and uncertainty intolerance on ego depletion. Uncertainty intolerance and perceived environmental uncertainty may have an interactive effect on ego depletion. Individuals with high uncertainty intolerance have an attentional bias toward the environment, are more sensitive to environmental uncertainty, and perceive it as threatening and intolerable (Freeston et al., 2020), try to avoid uncertainty, and seek to escape from complexities, new things, and ambiguous structures. In contrast, individuals with low uncertainty intolerance are generally more optimistic, happy-go-lucky, confident, and adventurous. They embrace uncertainty, regard it as challenging, desirable, and helpful, and do not attempt to artificially remove ambiguities and contradictions (Huang and Hu, 2021). Therefore, in high perceived environmental uncertainty situations, individuals with high uncertainty intolerance will experience a strong loss of self-control, resulting in anxiety, depression, anger, and other negative emotions, and to choose negative coping strategies (Zhang et al., 2020), thus leading to ego depletion. However, the individual with low uncertainty intolerance would adopt more positive coping styles and experience less ego depletion. Moreover, in low perceived environmental uncertainty situations, people have a high sense of control and security, and uncertainty intolerance is not associated with ego depletion. Therefore, we hypothesized that the interaction term of uncertainty intolerance and perceived environmental uncertainty was significantly associated with ego depletion, uncertainty intolerance was positively associated with ego depletion when in the high perceived environmental uncertainty situation.

### 1.4 The mediating role of negative coping styles in a moderating model

It is necessary to further explore the influence mechanism of uncertainty intolerance and perceived environmental uncertainty on ego depletion. Individual coping styles may be an important mediator in explaining the influence path. Coping styles are regarded as cognitive or behavioral strategies adopted by individuals when facing stressful situations (Thomas et al., 2011). It can be divided into two types: positive coping styles and negative coping styles. Positive coping styles include positive reappraisal of the situation, problem-focused coping, creation of positive meaning, finding growth potential, and seeking emotional and instrumental social support. Negative coping styles include self-blame, emotion-focused strategies, denial, vigilant coping, and cognition avoidance (Liu et al., 2016; Sun et al., 2019; Wang and Wang, 2019). Denial refers to refusing to acknowledge the existence or influence of stressors, vigilant coping refers to an intensified intake and processing of threatening information, and cognitive avoidance refers to turning away from threat-related cues (Grenier et al., 2005). Individuals with positive coping styles initiatively encounter stressors and positively solve problems; individuals with negative coping styles are unwilling to initiatively solve problems (Lentz et al., 2016), this is believed to occur as a means for individuals to protect their remaining resources rather than consume them (Zhang et al., 2020). The pressure-coping model highlights that when individuals face great pressure and lack internal and external resources to cope with this pressure, they will activate self-defense mechanisms and adopt negative coping strategies (Wills et al., 2001).

#### 1.4.1 The association between uncertainty intolerance, perceived environmental uncertainty, and negative coping styles

Perceived environmental uncertainty and uncertainty intolerance may affect coping styles. Previous research has demonstrated that perceived environmental uncertainty increases negative coping styles and impulsive choice strategies (Burk and Averbeck, 2023). Uncertainty management theory emphasizes that individuals first evaluate environmental uncertainty and that perceptions and evaluations of environmental uncertainty have a direct effect on individual psychology and behavior (Brashers, 2001). In high perceived environmental uncertainty situations, people with high uncertainty intolerance interpret this situation as threatening and intolerable, leading to anxiety and stress. Anxiety and stress would further induce people to apply more negative coping styles: (1) Anxiety drove decision-makers to adopt quick strategies to solve immediate problems regardless of long-term benefits, place greater emphasis on anecdotal information that was subjective and heuristic and ignore statistical information that was objective and factual, thus impacting decision-making capacity (Yang et al., 2015). (2) Anxious individual concern about a potential threat and negative future outcome, will stimulate pessimistic evaluations of decision-making events (Hartley and Phelps, 2012; Yang et al., 2015), and apply negative coping styles. (3) Anxiety could evoke high levels of autonomic arousal, impairing working memory capacity and executive function (Darke, 1988; Yang et al., 2015), leading people to apply negative coping styles. However, for those with low uncertainty intolerance, environmental uncertainty is perceived as acceptable, so they are more likely to adopt positive coping styles (Birrell et al., 2011). Based on these findings, we hypothesized that the interaction term of perceived environmental uncertainty and uncertainty intolerance was significantly associated with negative coping styles. In a high perceived uncertain environment, uncertainty intolerance was positively associated with negative coping styles.

#### 1.4.2 The relationship between negative coping styles and ego depletion

Negative coping styles are positively associated with ego depletion. Previous studies have demonstrated that negative coping styles hurt individual mental health and that they correlate with death anxiety among older adults (Liu et al., 2022), mobile phone dependence among adolescents (He et al., 2020), and work–family conflict among employees (Zhan et al., 2022), as well as with life satisfaction (Liu et al., 2016), and mental health status (Liu and Xin, 2022). Individuals who adopt negative coping styles tend to avoid or shelve problems, which leads to problems cannot be solved, further inducing greater pressure and anxiety, and decreasing self-control, resulting in greater ego depletion (Han et al., 2016).

### 1.5 Current study

**Figure 1:**
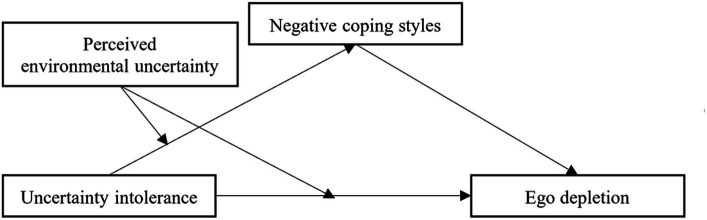
The hypothesized model of uncertainty intolerance associated with ego depletion.

This study focused on examining the association between uncertainty intolerance, perceived environmental uncertainty, and ego depletion, and the mediating role of negative coping styles in the moderating model. We focused on freshmen, the university is enclosed management during the COVID-19 pandemic, which is detrimental to students’ mental health and leads to ego depletion. Based on the review above, we hypothesized that the interaction term of uncertainty intolerance and perceived environmental uncertainty was significantly associated with negative coping styles and ego depletion, and negative coping styles would be positively associated with ego depletion (see Figure 1).

## 2 Methods

### 2.1 Participants

We recruited participants through a college student mental health course at a university located in Wuhan in November 2021. At this time, the epidemic prevention and control policy in China required people infected with COVID-19 to be isolated for treatment. We randomly selected three classes to recruit participants, with 150 students in each class. After reading and signing the informed consent, the participants voluntarily scanned the QR code on the poster to complete the electronic questionnaire survey. A total of 303 college students voluntarily participated in the research. Eleven participants were deleted because they gave up midway or their answers were highly consistent, leaving 292 valid data, including 173 males (59.20%) and 119 females (40.80%). Their age ranged from 16 to 20 (*M* = 18.23, *SD* = 0.59). In terms of their subjective family economic situation, 9 participants (3.10%) were very poor, 47 (16.10%) were a little poor, 221 (75.70%) were medium, and 15 (5.10%) were a little rich. G-power was used to calculate the number of participants with linear multiple regression, α = 0.05, 1-β = 0.95, effect size = 0.15, and the number of predictors is 3, the required number is 119.

### 2.2 Procedure

Approval to conduct this study was obtained from the university’s ethics committee. We recruited participants through a college student mental health course. To standardize the data collection process, two trained research assistants introduced this investigation in accordance with the procedure manual. Participants were informed that their answers would be anonymous and were reassured that they could withdraw from the study at any time without penalty. After receiving a briefing on the study, the participants provided informed consent. Students who volunteered to participate scanned the QR code to obtain an online questionnaire. They took approximately 10 min to complete all the questionnaires. The participants received partial credit for the course requirements.

### 2.3 Research material

Perceived environmental uncertainty: We used the dynamic work environment scale adopted by De Hoogh et al. (2005) to measure the uncertainty of the living environment. We have translated this questionnaire into Chinese, and have revised these items to ensure that they are suitable for measuring living environment uncertainty. It included three items. “What is the extent of challenge in your living environment?” “To which degree is your living environment dynamic?” “To what extent does your living environment offer great opportunities for change?” The participants responded to these items on a 5 Likert scale (1 = not at all, 5 = very much so). A higher score indicated greater perceived environmental uncertainty. This scale’s internal consistency was good in the current sample (Cronbach’s alpha = 0.75). We used exploratory factor analysis (EFA) and confirmatory factor analysis (CFA) to test the construct validity of this scale. Firstly, the Kaiser–Meyer–Olkin (KMO) sampling adequacy measure was used to determine whether our data were suitable for EFA. The results showed that the KMO was 0.69 (above 0.50), thus, the EFA can be carried out (Nunes et al., 2020). Secondly, we used principal component analysis to extract a factor, and the cumulative variance explanation rate was 67.30%, greater than 60%. The factor load of each item was 0.81, 0.85, and 0.80 respectively, all of them were higher than 0.5. Lastly, CFA was conducted, and the results showed that the degree of freedom was zero, and the chi-square statistic was zero. Consequently, the model was saturated (Steeger and Gondoli, 2013). In general, this scale had a good construct validity in the current sample.

Uncertainty intolerance: The uncertainty intolerance scale revised by Huang et al. (2017) was adopted in the current study to measure participants’ uncertainty intolerance, this scale included a total of 11 items. The participants responded to these items on a 5 Likert scale (1 = completely inconsistent with my idea, 5 = completely consistent with my idea). The higher the scale score indicated higher uncertainty intolerance. This scale’s internal consistency was good in the current sample (Cronbach’s alpha = 0.94).

Negative coping styles: The subscale of simple coping style was adopted to measure negative coping styles (Xie, 1998). This scale contained 20 items, and eight of them was related to negative coping styles, for example, “I relieve anxiety through smoking, drinking, taking drugs, and eating.” The participants responded to these items on a 4 Likert scale (0 = never, 3 = very frequently). The higher score indicated high negative coping styles. This scale’s internal consistency was good in the current sample (Cronbach’s alpha = 0.74).

Ego depletion: Simply ego depletion scale was used (Zhang et al., 2017), it included five items, for example, it takes a lot of work for me to focus on something. The participants responded to these items on a 7 Likert scale (1 = completely disagree, 7 = completely agree). A high score indicated high ego depletion. This scale’s internal consistency was good in the current sample (Cronbach’s alpha = 0.90).

Control variable: Previous research has shown that people’s negative coping styles may be influenced by people’s age (Chen, 2002), gender (Chen, 2002), and family socioeconomic status (Zhang, 2012). Thus, we measured age, gender, and family economic situation as the control variables in the current study.

### 2.4 Data analysis

Firstly, correlation analysis was carried out to analyze the correlations among all variables. The Pearson product difference correlation coefficient analyzed the strength and direction of the linear relationship between two variables. Secondly, we analyzed the relationship between uncertainty intolerance and ego depletion, the mediating role of negative coping styles, and the moderating role of perceived environmental uncertainty on the direct path and the first half of the mediating path. According to Hayes (2012), PROCESS programmed model 8 to estimate the indirect effect of the product of the independent variable (X) and the moderator (W) on the dependent variable (Y) through a mediator (M). Thus, in the current study, the PROCESS macro was used with model 8 to analyze this conditional process mode.

## 3 Results

### 3.1 Descriptive statistics and correlations among all variables

The correlations among all variables were calculated using Pearson’s product–moment correlation coefficient (see Table 1). The results indicated that uncertainty intolerance was significantly positively associated with perceived environmental uncertainty (*r* = 0.20, *p* < 0.01), negative coping styles (*r* = 0.17, *p* < 0.01), and ego depletion (*r* = 0.26, *p* < 0.01). Negative coping styles were positively associated with ego depletion (*r* = 0.23, *p* < 0.01). These correlation coefficients were all weak. The results showed that the higher an individual’s uncertainty intolerance, the higher the perceived environmental uncertainty, the more negative coping styles they would adopt, and the more ego depletion they would experience.

**Table 1:** Descriptive statistics and correlations among all variables.

	1. Gender	2. Age	3. Family economic situation	4. Perceived environment uncertainty	5. Uncertainty intolerance	6. Negative coping styles	7. Ego depletion
1	—						
2	0.03	—					
3	−0.02	−0.08	—				
4	−0.09	−0.10	0.06	—			
5	0.04	0.03	−0.08	0.20^**^	—		
6	−0.02	0.04	−0.05	−0.09	0.17^**^	—	
7	0.01	0.06	−0.13^*^	−0.05	0.26^**^	0.23^**^	—
*M*	0.59	18.23	2.79	9.64	30.62	19.16	14.66
*SD*	0.49	0.59	0.55	2.10	8.78	3.78	3.84

Gender was dummy variables, mean for gender is the percentage of male. ^*^p < 0.05, ^**^p < 0.01, ^***^p < 0.001.

The single-factor Harman test was used to assess the common method variance. The results of exploratory factor analysis showed that the first factor explained 21.51% of the variance (lower than the threshold of 40%), which indicated that the common method variance was not a serious threat in the current study.

### 3.2 The conditional process analysis

We used the PROCESS macro in SPSS (Hayes, 2018) to test the direct effect of uncertainty intolerance on ego depletion, the indirect effect via negative coping styles, and the moderating effect of perceived environmental uncertainty (see Figure 1). As the moderator (perceived environmental uncertainty) moderated the effect of the independent variable (uncertainty intolerance) on the mediator (negative coping styles) and the effect of the independent variable (perceived uncertainty intolerance) on the dependent variable (ego depletion), this conditional process model analysis was conducted using Model 8. All predictors were standardized to minimize multicollinearity. Moreover, the age, gender, and family economic situation were controlled in this model. Bootstrapping with 5,000 iterations was used to generate an approximation of the sampling distribution to obtain accurate confidence intervals.

First, in the model of independent and moderating variables correlated to the mediating variable, after controlling demographic variables, uncertainty intolerance was positively associated with negative coping styles (*B* = 0.18, *p* < 0.01), perceived environmental uncertainty was marginally significantly associated with negative coping styles (*B* = −0.11, *p* < 0.1), and their interactions term was positively significantly associated with negative coping styles (*B* = 0.09, *p* < 0.05). Second, in the model of independent, moderating, and mediating variables correlated to the dependent variable, after controlling demographic variables, uncertainty intolerance, and negative coping styles were both positively associated with ego depletion (*B* = 0.24, *p* < 0.001; *B* = 0.17, *p* < 0.01). Perceived environmental uncertainty and interactions term between perceived environmental uncertainty and uncertainty intolerance were not significantly associated with ego depletion (*B* = −0.07, *p =* 0.22; *B* = −0.003, *p* = 0.95), see Table 2.

**Table 2:** The results of conditional process analysis.

	Predictor variables	*R*	*R^2^*	*F*	*B*	*t*	LLCI	ULCI
Negative coping styles	Age	0.24	0.06	2.91^**^	0.02	0.19	−0.17	0.21
	Gender				−0.10	−0.88	−0.34	0.21
	Family economic situation				−0.05	−0.43	−0.25	0.16
	Uncertainty intolerance				0.18	2.99^**^	0.06	0.29
	Perceived environment uncertainty				−0.11	−1.77	−0.22	0.01
	Interaction term				0.09	2.03^*^	0.003	0.18
Ego depletion	Age	0.35	0.12	5.61^**^	0.06	0.59	−0.13	0.24
	Gender				−0.01	−0.04	−0.23	0.22
	Family economic situation				−0.18	−1.78	−0.38	0.02
	Uncertainty intolerance				0.24	4.15^***^	0.13	0.36
	Negative coping styles				0.17	3.03^**^	0.06	0.29
	Perceived environment uncertainty				−0.07	−1.23	−0.19	0.04
	Interaction term				−0.003	−0.06	−0.09	0.09

^*^P < 0.05, ^**^P < 0.01, ^***^P < 0.001.

The moderating role of perceived environmental uncertainty in the relationship between uncertainty intolerance and negative coping styles was significant. The simple slope analysis indicated that, in the low perceived environmental uncertainty condition, uncertainty intolerance was not significantly associated with negative coping styles. Whereas, in the high perceived environmental uncertainty condition, uncertainty intolerance was positively significantly associated with negative coping styles.

We also analyzed whether the perceived environmental uncertainty moderated the mediation effect. The results showed that perceived environmental uncertainty significantly moderated the mediation effect of negative coping styles in the relationship between uncertainty intolerance and ego depletion. In the low perceived environmental uncertainty condition, the mediation role of negative coping styles in the relationship between uncertainty intolerance and ego depletion was not significant (*B* = 0.01, LLCI=-0.03, ULCI=0.06). In the high perceived environmental uncertainty condition, the mediation role of negative coping styles in the relationship between uncertainty intolerance and ego depletion was significant (*B* = 0.05, LLCI=0.002, ULCI=0.11).

## 4 Discussion

This study aimed to explore the association between uncertainty intolerance, perceived environmental uncertainty, and ego depletion in early adulthood, and the mediating role of negative coping styles in the moderating model. Compared with previous studies, our study has the following contributions: First, we pay attention to the relationship between the interaction term of perceived environmental uncertainty and uncertainty intolerance and ego depletion, and the results showed that uncertainty intolerance was positively associated with ego depletion only in a high perceived uncertain environment. Secondly, we further pay attention to the mediating role of negative coping styles, and the results showed that uncertainty intolerance was positively associated with negative coping strategies only in a high perceived uncertain environment, which would further lead to ego depletion. Our study indicated that, compared with perceived environmental uncertainty, participants’ cognition toward environmental uncertainty would be much more associated with an individual’s coping styles and psychological state. Thirdly, the study was conducted during the COVID-19 pandemic, this is a special uncertain environment. People in Wuhan wear masks outside every day and often do nucleic acid testing. People infected with COVID-19 need to be isolated treated, and people who encounter an infector should be quarantined and observed. Therefore, at that time, people feel high environmental uncertainty, and their sense of control may decline. In the current study, we divided participants into high perceived environmental uncertainty and low perceived environmental uncertainty, but in fact, all participants might have a higher environmental uncertainty in that period compared with other periods. Fourth, we are focusing on freshman students in early adulthood (Collins and Madsen, 2006), the university is enclosed management during the epidemic period, which might be detrimental to students’ mental health and lead to high ego depletion.

Our results showed that uncertainty intolerance had a direct effect on ego depletion and an indirect effect on ego depletion through the mediating role of negative coping styles. Lentz et al. (2016) indicated that negative coping styles mainly include involuntary engagement (e.g., intrusive thoughts), voluntary disengagement (e.g., denial), and involuntary disengagement (e.g., emotional numbing). Thus, negative coping styles tend to avoid or shelve problems and do not address problems caused by uncertainty intolerance. Instead, negative coping styles induce anxiety and stress (Liu et al., 2022; Feng et al., 2023), and decrease life satisfaction (Liu et al., 2016), and mental health status (Liu et al., 2022). Moreover, it has been demonstrated that negative coping styles were associated with high levels of co-rumination, which refers to the excessive and repeated discussion of personal problems with another person while focusing almost exclusively on the negative feelings that these problems have elicited (Lentz et al., 2016; Yu et al., 2020). Co-rumination consumes cognitive resources, leading to ego depletion and fewer self-control resources (Hahm, 2011).

In addition, we found that the interaction term of uncertainty intolerance and perceived environmental uncertainty was significantly associated with negative coping styles. Previous findings have indicated that, when faced with an uncertain environment, individuals’ perception and assessment of uncertainty influence their coping styles (Carleton, 2016). Uncertain environment include uncertainty in knowledge of the environment, uncertainty about the intention of other people and organizations, and uncertainty about appropriate value judgment (Samsami et al., 2015). For individuals with high uncertainty intolerance, these uncertain environments lead them to experience high anxiety (Mallett et al., 2021; Yang and Li, 2023). Anxiety would limit cognitive ability and self-control, and the inability to use existing information to choose appropriate coping styles (Darke, 1988; Yang et al., 2015). Meanwhile, anxiety would increase an individual’s motivation to quickly reduce uncertainty, and thus make decisions that focus on short-term profit and ignore long-term profit (Remmers and Zander, 2018). Furthermore, they may delay decisions until the removal of major uncertainty or apply a negative coping strategy to remove the uncertainty (Samsami et al., 2015). In contrast, individuals with low uncertainty intolerance would feel low anxiety and low resource consumption when encountering an uncertain environment (Mallett et al., 2021; Yang and Li, 2023). Individuals are more likely to use more effective measures to reduce uncertainty, such as, actively using learning mechanisms to keep pace with environmental change, making plans to control uncertainty by taking actions to secure their future, or ensuring that specific actions will be undertaken if certain potential future events occur (Samsami et al., 2015).

However, inconsistent with one of our hypotheses, the interaction term of perceived environmental uncertainty and uncertainty intolerance was not significantly associated with ego depletion. Only uncertainty intolerance was significantly associated with ego depletion, whereas perceived environmental uncertainty was not significantly associated with ego depletion. Perceived environmental uncertainty did not directly lead to ego depletion but attitudes toward environmental uncertainty directly influenced ego depletion. This finding highlights the importance of subjective evaluation toward an objective environment. College students would experience various negative emotions during the COVID-19 pandemic, with anxiety, fear, sadness, and helplessness being more prominent (Fu, 2020). However, it is worth noting that these emotions are not directly caused by the epidemic but rather by uncertainty intolerance (Xiong et al., 2021). When faced with an uncertain environment, individuals first study and evaluate environmental uncertainty (Bredemeier and Berenbaum, 2008). Regardless of whether the objective environment is uncertain, individuals with high uncertainty intolerance are more likely to perceive it as uncertain, threatening, and risky, and feel pressure and unease (Miranda et al., 2008), this could easily lead to individuals becoming fixated on worry and anxiety for a long time. Excessive psychological distress in response to early warning signs and long-term stress would undoubtedly result in subsequent psychological problems and increase ego depletion.

### 4.1 Limitations and unanswered questions

This study had several limitations. First, the data were collected during the period of the COVID-19 pandemic, which was an uncertain event that people faced. This event brought about changes in college students’ lives and learning styles, resulting in an extension of uncertainty. Therefore, our findings should be interpreted within this context. Second, the self-report survey method would make our results affected by social desirability, and future research can investigate the uncertainty intolerance and coping styles through participants’ parents and friends. Third, our data were collected through cross-sectional surveys, and causality could not be inferred. Experimental and longitudinal studies are recommended. Specifically, future research could manipulate perceived environmental uncertainty through recall tasks, and manipulate uncertainty intolerance through role-playing paradigm in the laboratory to explore the causality. Moreover, future research can conduct two follow-up surveys at an interval of 6 months (time points 1 and 2 are represented by T1 and T2) to measure individuals’ uncertainty intolerance, perceived environmental uncertainty, negative coping styles, and ego depletion, and analyze the effect of uncertainty intolerance (T1), perceived environmental uncertainty (T1) on the negative coping styles (T2) and ego depletion (T2). Fourth, our current study divided participants into high uncertainty intolerance group and low uncertainty intolerance group from the one-dimensional perspective (Buhr and Dugas, 2002), future studies can use a two-dimensional perspective to divide uncertainty intolerance into anticipatory and inhibited types (Carleton et al., 2007). Fifth, we revised the work uncertainty scale adopted by De Hoogh et al. (2005) to measure perceived environmental uncertainty, which includes only three items and has never been used in Chinese samples. Perceived environmental uncertainty has two dimensions: perceived environmental harshness and perceived unpredictability. Future research should focus on these two dimensions, and developing a more rigorous and scientific environmental uncertainty questionnaire. Sixth, it is necessary to further explore the mechanism of uncertainty intolerance affecting ego depletion. Previous research has demonstrated that uncertainty intolerance may increase negative emotions and rumination, and decrease cognition resources (Mallett et al., 2021; Yildiz and Eldeleklioglu, 2021; Yang and Li, 2023), future studies could further explore the mediation role of negative emotion, rumination, and cognition resource in the relationship between uncertainty intolerance and ego depletion.

### 4.2 Implication

This study confirmed the significance of managing uncertainty intolerance for mental health in early adulthood. It is important to develop a positive perception and understanding of uncertainty and cultivate positive coping styles. It could help us turn threats into challenges, and promote individuals’ social adaptation and life satisfaction. We proposed the following specific intervention strategies: Firstly, rectify unreasonable cognition toward uncertainty, and enhance the tolerance of uncertainty. Uncertainty is the normal state of life, and an uncertain environment is a source of both despair and hope. It is important to find a breakthrough in the crisis. How our future is affected by the uncertain environment depends on how we perceive and cope with the uncertain environment. Secondly, learn emotional regulation strategies (e.g., progressive muscle relaxation, mindfulness meditation, and landing techniques). The direct manifestation of intolerance of uncertainty is negative emotions. The improvement of emotional regulation ability can help us alleviate the negative impact of uncertainty intolerance. Thirdly, learn and apply positive coping styles when encountering environmental uncertainty. Family, community, and school should guide early adulthood to learn positive coping styles, understand the short-term and long-term shortcomings of negative coping styles, and be proficient in using positive coping styles to solve problems.

### 4.3 Conclusion

The interaction term of uncertainty intolerance and perceived environmental uncertainty was significantly associated with the negative coping styles of early adulthood. Only in the high perceived environmental uncertainty situations, uncertainty intolerance was positively associated with negative coping styles of early adulthood. Moreover, negative coping styles were positively associated with ego depletion.

## Data availability statement

The data are available with doi: 10.6084/m9.figshare.22882145, https://doi.org/10.6084/m9.figshare.22882145.v1.

## Ethics statement

The studies involving humans were approved by Ethics Committee of the Institution of Psychology, China University of Geosciences. The studies were conducted in accordance with the local legislation and institutional requirements. Written informed consent for participation in this study was provided by the participants or the participants’ legal guardians/ next of kin.

## Author contributions

JS: completed the study design, data collection, and data analysis and wrote the article. XL: participated in the data collection and wrote the article. Both authors contributed to the article and approved the submitted version.
